# Supplementary material for: Generative adversarial networks based skin lesion segmentation
Source: Sci Rep. 2023 Aug 18;13:13467. doi: 10.1038/s41598-023-39648-8 (PMC10439152; doi:10.1038/s41598-023-39648-8)
Supplement: Supplementary file 1 — Supplementary Information. [file 41598_2023_39648_MOESM1_ESM.pdf]

Supplementary Material :

Discriminator:

Supplementary Figure 1 : The discriminator architecture has a convolutional layer (C) kernel size of 4 × 4 and stride of 2 × 2

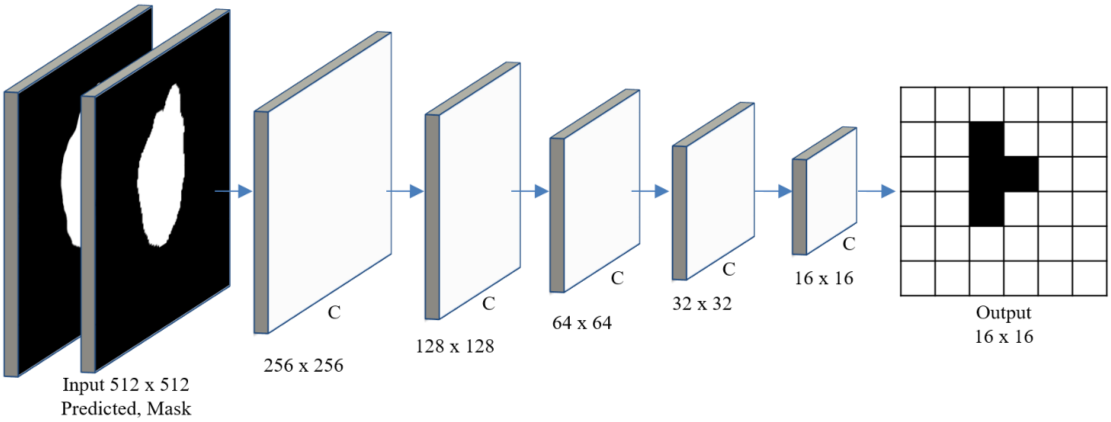

Evaluation Metrics :

The evaluation of segmentation is performed with various metrics like Dice Coefficient, Jaccard Score, Accuracy, Sensitivity
and Specificity which are described below:
Dice coefficient measures the overlap between label and predictions and is given as:

$$\frac{2 \times TP}{2 \times TP + FP + FN}$$

Jaccard Score computes the Intersection over Union (IoU) between the prediction and the ground truth as :

Pixel classification accuracy is $\frac{TP}{TP + FP + FN}$ calculated as:

$$\frac{TP + TN}{TP + FP + TN + FN}$$

Sensitivity estimates the level of actual positive values that are accurately recognized and given as:

$\frac{TP}{TP + FN}$ whereas, Specificity evaluates the percentage of negative values which are rightly identified and is obtained as:

$$\frac{TN}{FP + TN}$$

where T P : True Positive where the model correctly predicts the true class, T N : True Negative where the model correctly predicts the negative class, FP : False Positive model incorrectly predicts the positive class and FN : False Negative model incorrectly predicts the negative class.

Supplementary Table 1 : Performance of EGAN in terms of dice coefficient using i) dice loss and ii) morphology-based loss function is shown in Table 1 on ISIC 2018 test data accomplishes improved performance when trained with adversarial learning and morphology-based smoothing loss function.

**Loss Function**	**Dice Coefficient**
Dice loss	88.4
Morphology loss	90.1
